# mCRP as a Biomarker of Adult-Onset Still’s Disease: Quantification of mCRP by ELISA

**DOI:** 10.3389/fimmu.2022.938173

**Published:** 2022-07-01

**Authors:** Chitose Fujita, Yasuo Sakurai, Yuki Yasuda, Rino Homma, Cheng-Long Huang, Masaaki Fujita

**Affiliations:** ^1^ Division of Oncology, The Tazuke Kofukai Medical Research Institute, Kitano Hospital, Osaka, Japan; ^2^ The Japan-Multinational Trial Organization, Aichi, Japan; ^3^ Advanced Technology Research Department, Research and Development Center, Canon Medical Systems Corporation, Tochigi, Japan; ^4^ Division of Clinical Immunology and Rheumatology, Kansai Electric Power Hospital, Medical Research Institute, Osaka, Japan; ^5^ Department of Infectious Diseases, The Tazuke Kofukai Medical Research Institute, Kitano Hospital, Osaka, Japan

**Keywords:** modified-monomeric CRP, adult-onset Still’s disease, autoimmune diseases, biomarker, diagnosis

## Abstract

**Background:**

C-reactive protein (CRP) is a dynamic protein that undergoes conformational changes between circulating native pentameric CRP (pCRP), pentameric symmetrical forms (pCRP*) and monomeric (or modified) CRP (mCRP) forms. mCRP exhibits strong pro-inflammatory activity and activates platelets, leukocytes, and endothelial cells. Abundant deposition of mCRP in inflamed tissues plays a role in several disease conditions, such as ischemia/reperfusion injury, Alzheimer’s disease, and cardiovascular disease. Although pCRP is typically quantified rather than mCRP for clinical purposes, mCRP may be a more appropriate disease marker of inflammatory diseases. Therefore, simple methods for quantifying mCRP are needed.

**Methods:**

We developed a specific enzyme-linked immunosorbent assay (ELISA) to measure plasma levels of mCRP. Plasma mCRP concentration was measured in patients with adult-onset Still’s disease (AOSD) (n=20), polymyalgia rheumatica (PMR) (n=20), rheumatoid arthritis (RA) (n=30), infection (n=50), and in control subjects (n=30) using the developed ELISA.

**Results:**

We demonstrated that mCRP is elevated in some inflammatory autoimmune diseases, particularly AOSD. The mCRP concentration was also significantly higher among AOSD patients than RA, PMR patients and controls (477 ng/ml, 77 ng/ml, 186 ng/ml, and 1.2 ng/ml, respectively). Also, the mCRP (×1,000)/pCRP ratio was significantly higher among AOSD patients than RA, PMR, and infection patients (3.5, 0.6, 1,6, and 2.0, respectively).

**Conclusion:**

The plasma mCRP levels are elevated in some autoimmune diseases, particularly AOSD. The plasma mCRP levels may therefore be a potentially useful biomarker for AOSD.

## Introduction

C-reactive protein (CRP), an acute-phase reactant of the pentraxin family, is synthesized in the liver under conditions of inflammation and infection in response to certain cytokines (1, 2). The property as acute-phase reactant has long been exploited for clinical purposes. CRP is a dynamic protein that undergoes conformational changes between circulating native pentameric CRP (pCRP), pentameric symmetrical forms (pCRP*) and monomeric (or modified) CRP (mCRP) forms (3–10). It is thought that pCRP can dissociate into mCRP through pCRP* (8, 10, 11). The dissociation to pCRP* and mCRP causes to expose neoepitopes (8, 10). The dissociation can occur under some conditions including the presence of urea and/or the absence of calcium (12). *In vivo*, pCRP can dissociate into mCRP in a process involving several steps (10, 13). In the first step, the enzyme phospholipase A2 mediates the exposure of phosphocholine (PC) on the cell surface. pCRP then binds to the cell surface *via* interaction with PC. In the final step, cell-bound pCRP undergoes a conformational change that leads to its dissociation to mCRP. Although some research suggested that pCRP functions as a direct mediator of inflammation (14), others indicated that pCRP exhibits only weak anti-inflammatory activity (15). In contrast, numerous studies indicate that mCRP exhibits strong pro-inflammatory activity (2, 7, 8, 16–22). In addition to activating the complement system (21, 23), mCRP activates platelets, leukocytes, and endothelial cells (2, 7, 8, 13, 17). We previously reported that mCRP also activates monocytes *via* interaction with integrins (20). pCRP* also have pro-inflammatory functions (10).

Abundant deposition of mCRP in inflamed tissues plays a role in several disease conditions, such as ischemia/reperfusion injury, Alzheimer’s disease (24, 25), and cardiovascular disease (26, 27). Although pCRP is typically quantified rather than mCRP for clinical purposes, mCRP may be a more appropriate disease marker of inflammatory diseases. Indeed, microparticle-bound mCRP is reportedly a better diagnostic marker than pCRP for myocardial infarction (28) and vascular diseases (29). However, the procedure for quantifying microparticle-bound mCRP is complex and thus unsuited for routine clinical use. Therefore, simple methods for quantifying mCRP are needed. In a previous study, we screened >1800 anti-mCRP monoclonal antibody (mAb) clones and identified several mAbs that recognize mCRP but not pCRP (30). In the present study, we employed these antibodies to develop a specific enzyme-linked immunosorbent assay (ELISA) to measure plasma levels of mCRP. Furthermore, we demonstrated that mCRP levels are elevated in some inflammatory autoimmune diseases. The quantification of plasma mCRP may be useful for differential diagnosis of diseases.

## Materials and Methods

### Ethical Approval

The study protocols were approved by the Institutional Ethics Committee of the Tazuke Kofukai Medical Research Institute Kitano Hospital (protocol approval number: 1704009) and Kansai Electric Power Hospital (protocol approval number: 20-022).

### Patients and Controls

A total 70 patients with newly diagnosed autoimmune diseases in active phase, 50 patients with infection, and 30 healthy controls were enrolled (Tazuke Kofukai Medical Research Institute, Kitano Hospital, Osaka, Japan, or Kansai Electric Power Hospital, Osaka, Japan). The group of patients with autoimmune diseases consisted of 20 patients with adult-onset Still’s disease (AOSD), 20 patients with polymyalgia rheumatica (PMR), and 30 patients with rheumatoid arthritis (RA). AOSD patients fulfilled Yamaguchi’s diagnostic criteria for AOSD (31). PMR patients fulfilled the 2012 Provisional Classification Criteria for Polymyalgia Rheumatica, a European League against Rheumatism/American College of Rheumatology collaborative initiative (32). RA patients fulfilled the 1987 or 2010 American College of Rheumatology classification criteria (33, 34). Infection was diagnosed based on clinical symptoms, microbiological or radiographic methods, and good response to antibiotics. Individuals undergoing routine check-up with no history of rheumatic or infectious diseases were enrolled as controls. In addition, we collected time-course blood samples in 9 AOSD patients.

### Generation and Selection of mAbs

mAbs against mCRP were generated and selected as previously described (30). Briefly, mCRP were generated from commercially available human pCRP (human pleural fluid, Lee BioSolutions, Maryland Heights, MO, USA) as described previously (34). BALB/c mice were injected intraperitoneally with approximately 50 μg of mCRP. Spleen cells or lymphoid tissues were fused with mouse myeloma cells (X63Ag8.653, ATCC). A total of 1,824 hybridoma culture supernatants were harvested, and their reactivity against mCRP was estimated by ELISA. A total of 36 positive hybridoma clones were selected to obtain antibodies that strongly bound to mCRP. Using an inhibition ELISA, a total of 6 antibodies (3C, 12C 18A 19C, 21A, 35A) that specifically reacted with mCRP were selected. The specificity against mCRP was demonstrated by inhibition ELISA in our previous study (30).

### Antibody Combination

Antibody combination was screened by assessing the reactivity to mCRP and cross-reactivity to pCRP. Briefly, 96-well culture plates (NUNC, Thermo Fisher) were coated with 10 μg/mL of anti-mCRP mAb (3C, 12C, 18A, 19C, 21A, or 35A) in 0.1 M carbonate-bicarbonate buffer, pH 9.5 (Immuno-Biological Laboratories Co., Ltd. Japan (IBL)) and incubated overnight at 4°C. After saturation of free plastic sites of the wells with 1% BSA in PBS overnight at 4°C, 100 μl of PBS with 1% BSA containing mCRP or pCRP was added to the wells and incubated for 60 min at 37°C. Unbound mCRP/pCRP was removed by rinsing the wells four times, and bound mCRP or pCRP was detected using 1 μg/mL of biotin-conjugated anti-mCRP mAb (3C, 12C, 18A, 19C, 21A, or 35A) and HRP-conjugated avidin (Vector Laboratories) and peroxidase substrates (*o*-Phenylenediamine dihydrochloride (OPD), Sigma-Aldrich). After stopping the reaction with H_2_SO_4_, absorbance of the optical density at 490 nm (OD_490_) of the wells was measured using a microplate reader (Molecular Device, Emax) Overall, 12C/18A was selected as the best combination.

### Generation of mCRP Assay Kit and Plasma mCRP Quantification

In antibody combination test, 12C/18A was selected as the best combination. Next, mCRP sandwich ELISA kit was developed by using these two antibodies (IBL). For this purpose, anti-mCRP mAb (18A) was labeled with HRP. Blood samples for plasma mCRP analysis were collected using EDTA-K2. Plasma mCRP concentration was measured by using the sandwich ELISA. Briefly, 96-well culture plates (NUNC, Thermo Fisher) were coated with 2 μg/mL of anti-mCRP mAb (12C) and incubated overnight at 4°C. After saturation of free plastic sites of the wells with 1% BSA in PBS overnight at 4°C, 100 μl of PBS with 1% BSA containing diluted plasma was added to the wells and incubated for 60 min at 37°C. Unbound mCRP were removed by rinsing the wells four times, and bound mCRP was detected using 0.4 μg/mL of HRP-conjugated anti-mCRP mAb (18A) and peroxidase substrates (3,3’,5,5’-Tetramethyl benzidine, IBL). After stopping the reaction with 1N H_2_SO_4_, the optical density at 450 nm (OD_450_) and 650 nm (OD_650_) of the wells was measured using a microplate reader (Molecular Device, Emax). mCRP concentration was calculated based on the value of OD450-650 nm using standard curve.

### pCRP Quantification

pCRP was measured by the latex agglutination methods as routine clinical laboratory tests (N-assay LA CRP-T or N-assay LA CRP-S D-type (Nittobo Medical Co., Ltd., Japan).

### Statistical Analysis

Treatment differences were evaluated using a Kruskal-Wallis test followed by Dunn’s post-test comparison for plasma mCRP concentration in each disease group or Wilcoxon signed-rank test for time course changes in plasma mCRP concentration. Receiver operating characteristic (ROC) curves were constructed to assess the analytical performance of plasma mCRP to diagnose AOSD. To determine the cut-off value, the point on the ROC curve with the minimum distance from the left-upper corner of the unit square was selected. Statistical analyses were performed using Prism 6.0 software (GraphPad Software).

## Results

### Analytical Performance of mCRP ELISA Kit

The mCRP detection sensitivity was 0.07 ng/mL. The measurement range for the mCRP assay was approximately 0.63-40 ng/mL. Standard curve and measured values were shown in **Supplemental Figure 1**. The cross-reactivity for pCRP was <0.1%. Dilution linearity of plasma samples was certified (*R^2 =^* 1; 5 to 50 ng/ml) (**Supplemental Figure 1**). The measurement range was determined by 2-fold serial dilution methods (mCRP: maximal concentration: 400 ng/ml). The cross reactivity for pCRP was determined by measuring excess amounts of pCRP more than 1000 times of mCRP concentrations. The within-run reproducibility coefficient of variation (CV) values for plasma mCRP ranged from 3.5% to 5.2% (n=24), and the between-run reproducibility CV values ranged from 2.0% to 8.9% (n=4).

### Patient Characteristics

The characteristics of the patients enrolled in the study are presented in **Table 1** (e.g., age, sex, white blood cell [WBC] count, pCRP, mCRP). The infection group included 20 patients with pneumonitis, 23 with urinary tract infection, 2 with infective endocarditis, 3 with pharyngitis, 1 with kidney cyst infection, and 1 with cellulitis. No significant difference in gender was observed between the AOSD, RA, PMR, infection, and control groups. Age was significantly higher in the PMR group than AOSD and control groups. pCRP level was higher in the AOSD, RA, PMR, and infection groups compared with the control group. In addition, pCRP was significantly higher in the infection group than RA group. WBC count was higher in the AOSD, PMR, and infection groups compared with the control group. In addition, WBC count was significantly higher in the AOSD group than the RA group.

**Table 1:** Patient characteristics.

Variable	AOSD	RA	PMR	Infection	Controls	p-value
	n = 20	n = 30	n = 20	n = 50	n = 30	
Age years	57.8(23-87)	64.4(20-84)	75(41-84)	65.6(23-89)	54(42-72)	0.44
Female; male	12; 8	16; 14	11; 9	26; 24	16; 14	0.94
WBC/μl	14185±4892	10524±2866	10875±3469	10251±4883	5879±1632	<0.05
pCRP mg/dl	14.9±9.3	7.9±7.9	12.2±6.6	17.4±9.9	0.05±0.04	<0.05
mCRP ng/ml	477(100, 2570)	77(0.035,501)	77(0.035,626)	228(0.035,1086)	1.2(0.035,10)	<0.05

p: AOSD with control.

Patient’s baseline parameters were shown as mean±standard deviation or median with range.

### Increased Plasma mCRP in AOSD Patients

**Figure 1:**
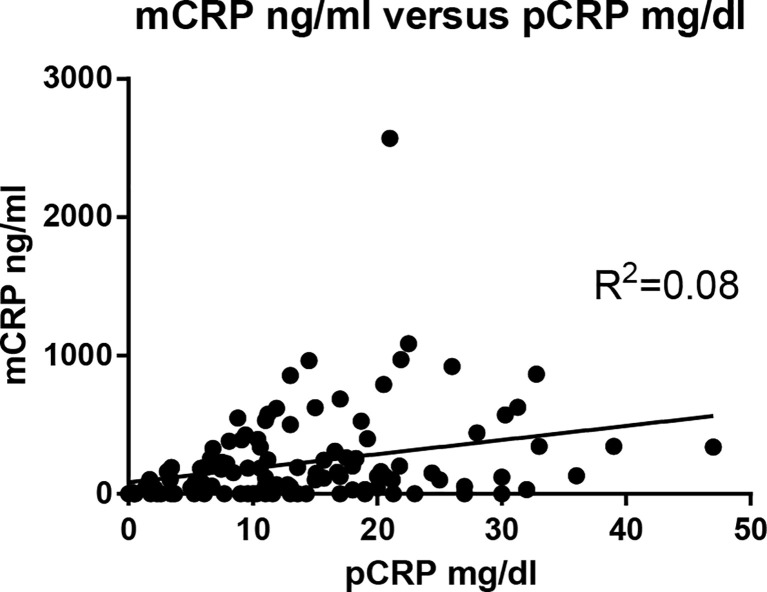
Correlation between mCRP and pCRP concentrations. The correlation between mCRP and pCRP concentration was calculated. Although high-pCRP samples tended to exhibit high mCRP levels, no positive correlation was observed (*R^2 =^* 0.08).

**Figure 2:**
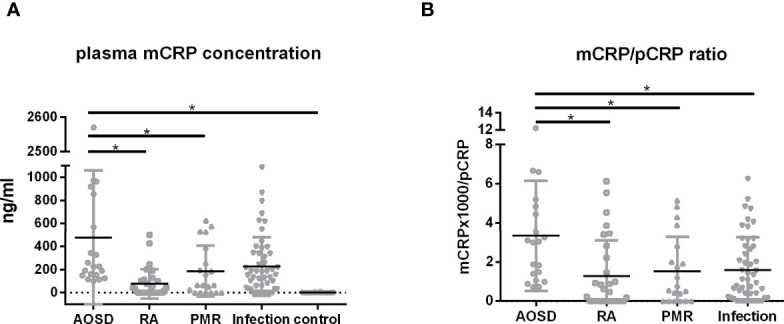
Plasma mCRP concentration. Plasma mCRP concentration was measured in patients with AOSD (n = 20), PMR (n = 20), RA (n = 30), infection (n = 50), and in control subjects (n = 30) using the developed ELISA. **(A)** Plasma mCRP concentration in each group. **(B)** mCRP (×1,000)/pCRP ratio. Statistical analysis was performed using a Kruskal-Wallis test followed by Dunn’s post-test comparison. Data are shown as means +/- SD. *P < 0.05.

**Figure 3:**
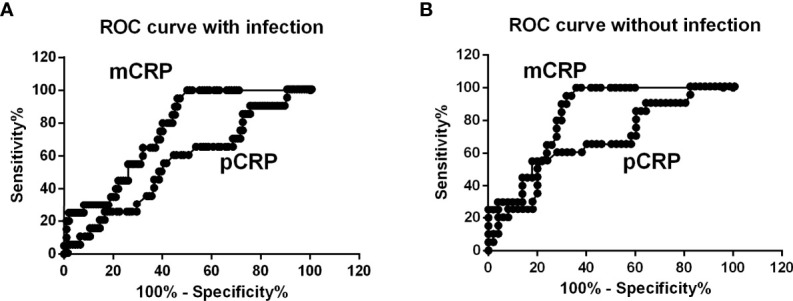
ROC curve analysis. **(A)** ROC curves were analyzed to determine diagnostic value of plasma mCRP level for distinguishing AOSD patients from those with RA, PMR, and infection. The area under the curve for mCRP was 0.76 (95% CI, 0.66 to 0.85) (*P = *0.0003). **(B)** ROC curve analysis with exclusion of infection patients. For the diagnostic value of plasma mCRP level for distinguishing AOSD patients from those with RA or PMR and controls, the area under the curve for mCRP was 0.83 (95% CI, 0.74 to 0.92) (*P* < 0.0001). To determine the cut-off value, the point on the ROC curve with the minimum distance from the left-upper corner of the unit square was selected.

The correlation between mCRP and pCRP levels was calculated. Although high-pCRP samples tended to exhibit high mCRP levels, no positive correlation was observed (*R^2 =^* 0.08) (**Figure 1**). The distribution of mCRP concentration among the enrolled patients is shown in **Figure 2A**. mCRP levels were significantly higher in the AOSD, RA, and PMR groups than in the control group (477, 77, and 186 vs 1.2 ng/ml, respectively). Notably, mCRP levels were significantly higher in AOSD patients than in RA or PMR patients. Interestingly, mCRP levels were also significantly higher in AOSD patients than in acute myocardial infarction patients reported by Wang (477 ng/ml vs 20.96 ng/ml) (35). In addition, mCRP levels of AOSD patients in the present study were higher than those of patients with skin-related autoimmune diseases reported by Zhang (eczema: 30.0 ng/ml, psoriasis: 35.4 ng/ml, urticaria: 59.8 ng/ml) (36) and those of patients with osteoarthritis reported by Liang (osteoarthritis:12.5 ng/ml) (37). mCRP levels were also significantly higher in the infection group than in the control group in the present study (228 ng/ml vs 1.2 ng/ml, respectively). However, one patient in AOSD groups showed the very high mCRP concentration (2570 ng/ml). For this patient (49 years old female), pCRP was 21 mg/dl, WBC was 14700/μl, ferritin was 8200 ng/ml. Also, one patient in infection groups showed the very high mCRP concentration (1086 ng/ml). For this patient (76 years old male, urinary tract infection), pCRP was 22.5 mg/dl, WBC was 6800/μl, ferritin was 1256 ng/ml. These highest values may increase the real averaged plasma mCRP concentration. Furthermore, mCRP levels were >100 ng/ml in all 20 AOSD patients. In contrast, mCRP levels were >100 ng/ml in only 7 of 30 RA and 9 of 20 PMR patients. The mCRP (×1,000)/pCRP ratio was also significantly higher among AOSD patients than RA, PMR, and infection patients (3.5, 0.6, 1,6, 2.0, respectively) (**Figure 2B**). ROC curves were analyzed to determine the diagnostic value of plasma mCRP level for discriminating AOSD patients from the RA, PMR and infection groups. The area under the curve for mCRP was 0.76 (95% confidence interval [CI], 0.66 to 0.85) (P=0.0003). At the optimal cut-off value of 188.0 ng/ml, the sensitivity and specificity were 65% and 68%, respectively (**Figure 3A**). On the other hand, the area under the curve for pCRP was 0.55 (95% confidence interval [CI], 0.42 to 0.69) (P=0.46). At the optimal cut-off value of 12.9 mg/dl, the sensitivity and specificity were 60% and 56%, respectively (**Figure 3A**). An ROC curve analysis was also conducted with exclusion of the infection groups. For the diagnostic value of plasma mCRP level for discriminating AOSD patients from the RA and PMR, the area under the curve for mCRP was 0.83 (95% CI, 0.74 to 0.92) (*P*<0.0001). At the optimal cut-off value of 138.5 ng/ml, the sensitivity and specificity were 80% and 72%, respectively (**Figure 3B**). On the other hand, the area under the curve for pCRP was 0.66 (95% CI, 0.52 to 0.80) (P=0.036). At the optimal cut-off value of 13.6 mg/dl, the sensitivity and specificity were 55% and 78%, respectively (**Figure 3B**).

### Time Course of Changes in Plasma mCRP Level in AOSD Patients

**Figure 4:**
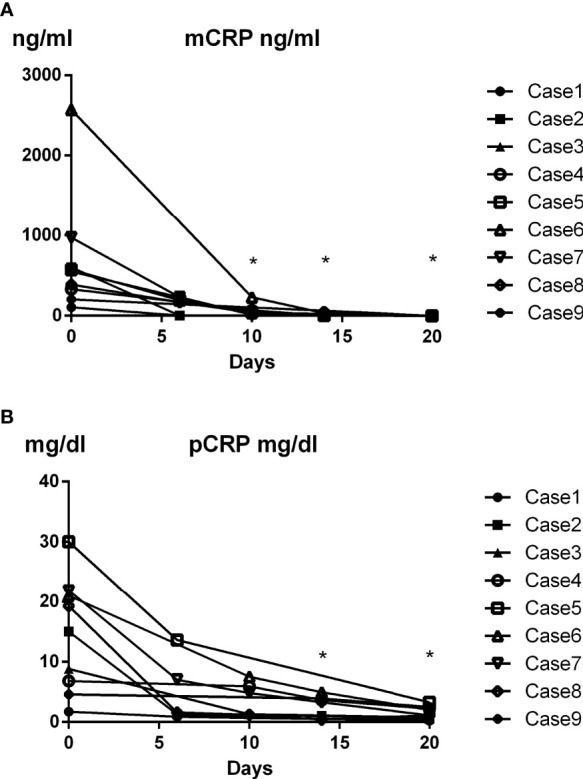
Time course of changes in plasma mCRP level in AOSD patients. Dynamic changes in plasma mCRP concentration in AOSD patients after immunosuppressive therapy were evaluated (n = 9) at the indicated time points. Plasma mCRP levels decreased more rapidly than pCRP levels following immunosuppressive therapy. Statistical analysis was performed using a Wilcoxon signed-rank test. *P < 0.05. mCRP concentration on day 0 and indicated days were compared.

Dynamic changes in plasma mCRP concentration in AOSD patients were evaluated after immunosuppressive therapy. Interestingly, plasma mCRP levels declined more rapidly than pCRP levels following immunosuppressive therapy (**Figures 4A, B**). mCRP became undetectable before pCRP, in accordance with the current hypothesis (3).

## Discussion

### Comparison With Previous Developed mCRP ELISA

In comparison with previous developed mCRP ELISA, the measuring range for mCRP assay kit developed by Wang (35) is from 1.0 to 160 ng/ml. On the other hand, the measuring range of our mCRP assay kit is from 0.63 to 40 ng/ml and the mCRP detection sensitivity was 0.07 ng/mL. Also, for mCRP assay kit developed by Zhang (36), it seems that pCRP was detected at 1-10 μg/ml. On the other hand, the cross-reactivity for pCRP of our kit was <0.1%. The reason why there are some differences in the measuring range and the mCRP detection sensitivity remains to be elucidated. Different antibodies are used for the assay in each group and these differences may affect the measurement results.

### Serological Differential Diagnosis of Autoimmune Diseases

RA, PMR, and AOSD are the three most common systemic rheumatic diseases in adults. PMR occurs exclusively in individuals over the age of 50 years (38), in contrast to RA, which has a typical onset age of between 30 and 50 years (39). Onset of AOSD occurs most commonly in two age ranges, 16 to 25 and 36 to 46 years (40). However, other characteristic symptoms often overlap, making differential diagnoses difficult. Each condition can present with diffuse musculoskeletal pain, considerable stiffness, fever, and fatigue. Laboratory test results are also very similar, with all cases showing modest anemia, thrombocytosis, elevated acute-phase reactants (including both the erythrocyte sedimentation rate and CRP). AOSD patients typically present with a salmon-pink rash, as well as with joint pain and high fever. However, 12-20% of AOSD patients lack a typical rash (41–43), which complicates the diagnosis of AOSD. In addition, negative test results for rheumatoid factor and anti-cyclic citrullinated peptide antibodies increase the difficulty of diagnosing RA. No specific marker has been identified for PMR. Therefore, it is important for physicians to serologically distinguish AOSD from seronegative RA or PMR. The identification and clinical development of a suitable biomarker is expected to enable clinicians to distinguish AOSD from seronegative RA or PMR.

In this study, we demonstrated that mCRP levels are significantly higher in AOSD, RA, and PMR patients compared with healthy individuals (477, 77, and 186 vs 1.2 ng/ml), and mCRP levels were significantly higher in AOSD patients than in RA or PMR patients (**Figure 2A**). The mCRP/pCRP ratio (×1,000) was significantly higher among AOSD patients than RA, PMR, and infection patients (3.5 vs 0.6, 1.6, and 2.0, respectively) (**Figure 2B**). Notably, the mCRP/pCRP ratio was significantly higher in AOSD patients than patient with infection and RA and PMR patients, although a slight increase in mCRP was confirmed in RA, PMR, and infection patients. Furthermore, ROC curves were analyzed to determine diagnostic value of plasma mCRP level for distinguishing AOSD patients from patients with RA or PMR, excluding patients with infection. For the diagnostic value of plasma mCRP level for distinguishing AOSD patients from RA or PMR, excluding patients with infection, the area under the curve for mCRP was 0.83 (95% CI, 0.74 to 0.92) (*P*<0.0001), and the sensitivity and specificity were 80% and 72%, respectively, at the optimal cut-off value of 138.5 ng/ml (**Figure 3B**). As expected, it was difficult to distinguish AOSD from RA, PMR and infection groups by means of the measurement of the pCRP levels.

As shown in **Figure 2**, the increase in plasma mCRP level was not specific to AOSD patients. In particular, a slight increase in mCRP level was confirmed in infection patients. However, in clinical settings, physicians can typically discriminate infection patients from non-infection patients, although this can be difficult in some cases. Thus, our results indicate that the plasma mCRP level and/or mCRP/pCRP ratio represent good diagnostic markers for AOSD, particularly in the case of patients with unsuspected infectious diseases. Previous studies have shown that serum ferritin levels are increased in AOSD (44). The measurement of both ferritin and mCRP (mCRP/pCRP ratio) could increase the accuracy of AOSD diagnosis. Thus, the quantification of plasma mCRP level and/or calculation of the mCRP/pCRP ratio represent convenient diagnostic tools for AOSD. Interestingly, in the present study, plasma mCRP levels deceased more rapidly than pCRP levels following immunosuppressive therapy, with mCRP becoming undetectable before pCRP (**Figure 4**). Although the sample size of this study was small, our results indicate that mCRP may be an early diagnostic marker for monitoring disease activity. This characteristic could be useful in helping clinicians make the right choice in terms of either increasing or decreasing immunosuppressive therapy.

The current study has several limitations. First, in this study, patients taking corticosteroids and/or immunosuppressive drugs were excluded from the study, in consideration of the effect of immunosuppressive drugs on measured values. In addition, other autoimmune diseases such as dermatomyositis, systemic lupus erythematosus, anti-neutrophil cytoplasmic antibody-associated vasculitis and/or large vessel vasculitis were not analyzed in this study. A larger-size cohort study including patients taking corticosteroids and/or immunosuppressive drugs and patients with the abovementioned autoimmune diseases is needed to address these issues. Second, the infection group mainly included patients with pneumonitis or urinary tract infections, which may have biased the results. Sub-analyses of every infectious disease types also remain to be addressed. Third, the rate of increase in plasma mCRP levels after disease onset was not analyzed in this study. To address this issue, the analysis of cases with recurrence of illness may be useful. Also, the reason why mCRP in particular elevated in AOSD remains to be elucidated. AOSD is systemic inflammatory diseases. On the other hand, RA and/or PMR are inflammatory diseases of joints. Therefore, the elevation of mCRP in bloods may reflect systemic inflammation.

In conclusion, the plasma mCRP levels are elevated in some autoimmune diseases, particularly AOSD. The plasma mCRP levels may therefore be a potentially useful biomarker for AOSD. Clinical measurement of mCRP in combination with other biomarkers such as ferritin might strengthen the diagnosis of AOSD. As the sample size in this study was small, a larger-size cohort study is needed to demonstrate mCRP as a novel biomarker for AOSD.

## Data Availability Statement

The original contributions presented in the study are included in the article/**Supplementary Material**. Further inquiries can be directed to the corresponding author.

## Ethics Statement

The study protocols were reviewed and approved by the Institutional Ethics Committee of the Tazuke Kofukai Medical Research Institute Kitano Hospital (protocol approval number: 1704009) and Kansai Electric Power Hospital (protocol approval number: 20-022). The patients/participants provided their written informed consent to participate in this study.

## Author Contributions

MF conceptualized the project. CF, YS, YY, RH, CL-H, and MF performed experiments and generated the data used in this manuscript. MF wrote the majority of this manuscript, with edits and review provided by all co-authors. All authors contributed to the article and approved the submitted version.

## Funding

This work was supported by funding to Masaaki Fujita from the Canon Medical Systems Corporation and the Japan Society for the Promotion of Science, Grant-in Aid for Scientific Research(19K08901).

## Conflict of Interest

Authors YY, YS and RH were employed by the company Canon Medical Systems Corporation. This study received funding from Canon Medical Systems Corporation. The funder was involved in the study design and the decision to submit it for publication. The remaining authors declare that the research was conducted in the absence of any commercial or financial relationships that could be construed as a potential conflict of interest.

## Publisher’s Note

All claims expressed in this article are solely those of the authors and do not necessarily represent those of their affiliated organizations, or those of the publisher, the editors and the reviewers. Any product that may be evaluated in this article, or claim that may be made by its manufacturer, is not guaranteed or endorsed by the publisher.

## Supplementary Material

Supplementary Figure 1 Analytical performance. The analytical performance of the mCRP assay kit was evaluated. **(A)** Measurement range for the mCRP assay was determined as approximately 0.63 to 40 ng/ml. **(B)** Dilution linearity of plasma samples was certified (*R^2 =^* 1; 5 to 50 ng/ml).
