# Supplementary figures and images for: mCRP as a Biomarker of Adult-Onset Still’s Disease: Quantification of mCRP by ELISA
Source: Front Immunol. 2022 Jul 1;13:938173. doi: 10.3389/fimmu.2022.938173 (PMC9284222; doi:10.3389/fimmu.2022.938173)

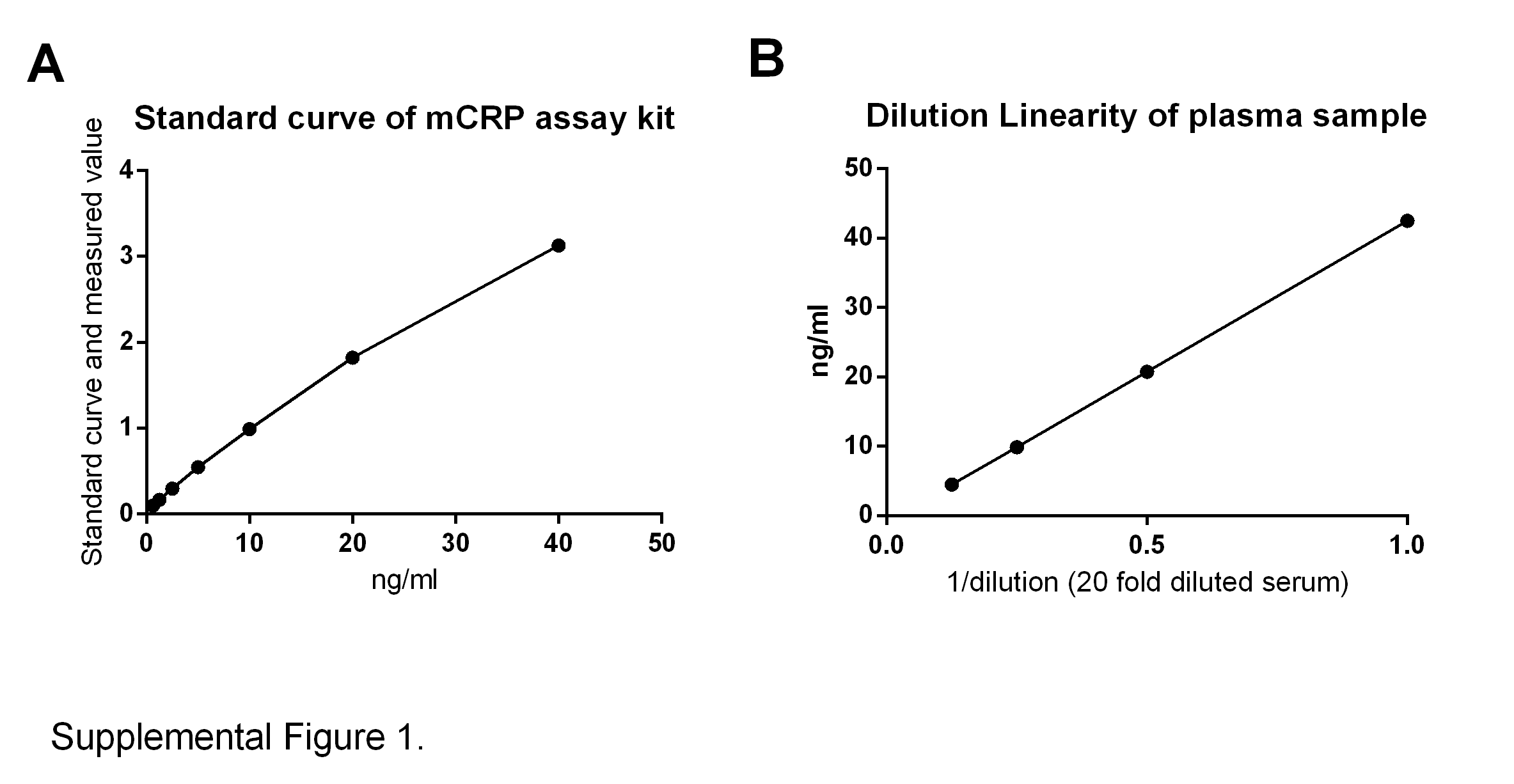

Supplement: Supplementary Figure 1 — Analytical performance. The analytical performance of the mCRP assay kit was evaluated. (A) Measurement range for the mCRP assay was determined as approximately 0.63 to 40 ng/ml. (B) Dilution linearity of plasma samples was certified (R2 = 1; 5 to 50 ng/ml). [file Image_1.tif]
